# Real-Time Observation of the Interaction between Thioflavin T and an Amyloid Protein by Using High-Sensitivity Rheo-NMR

**DOI:** 10.3390/ijms18112271

**Published:** 2017-10-28

**Authors:** Naoto Iwakawa, Daichi Morimoto, Erik Walinda, Yasushi Kawata, Masahiro Shirakawa, Kenji Sugase

**Affiliations:** 1 Department of Molecular Engineering, Graduate School of Engineering, Kyoto University, Kyoto-Daigaku Katsura, Nishikyo-ku, Kyoto 615-8510, Japan; iwakawa.naoto.46w@st.kyoto-u.ac.jp (N.I.); morimoto@moleng.kyoto-u.ac.jp (D.M.); shirakawa@moleng.kyoto-u.ac.jp (M.S.); 2 Department of Molecular and Cellular Physiology, Graduate School of Medicine, Kyoto University, Yoshida Konoe-cho, Sakyo-ku, Kyoto 606-8501, Japan; walinda.erik.6e@kyoto-u.ac.jp; 3 Department of Chemistry and Biotechnology, Graduate School of Engineering, Tottori University, 4-101 Koyama-cho Minami, Tottori 680-8552, Japan; kawata@bio.tottori-u.ac.jp

**Keywords:** amyloid fibrils, thioflavin T, molecular interactions, Rheo-NMR, real-time observation, SOD1

## Abstract

Amyloid fibril formation is associated with numerous neurodegenerative diseases. To elucidate the mechanism of fibril formation, the thioflavin T (ThT) fluorescence assay is widely used. ThT is a fluorescent dye that selectively binds to amyloid fibrils and exhibits fluorescence enhancement, which enables quantitative analysis of the fibril formation process. However, the detailed binding mechanism has remained unclear. Here we acquire real-time profiles of fibril formation of superoxide dismutase 1 (SOD1) using high-sensitivity Rheo-NMR spectroscopy and detect weak and strong interactions between ThT and SOD1 fibrils in a time-dependent manner. Real-time information on the interaction between ThT and fibrils will contribute to the understanding of the binding mechanism of ThT to fibrils. In addition, our method provides an alternative way to analyze fibril formation.

## 1. Introduction

Amyloid fibril formation inside cells is associated with various neurodegenerative diseases such as Alzheimer’s disease (AD), Parkinson’s disease (PD), and amyotrophic lateral sclerosis (ALS) [1]. The thioflavin T (ThT) fluorescence assay is one of the most typical methods used to analyze amyloid fibril formation [2,3]. ThT is a benzothiazole dye that exhibits fluorescence enhancement upon selective binding to amyloid fibrils [4]. The specific interactions between ThT and amyloid fibrils can be detected not only by fluorescence spectroscopy, but also by nuclear magnetic resonance (NMR) spectroscopy. A previous NMR study revealed characteristic ^1^H NMR signal changes of ThT upon association with fibrils and protofibrils [5], implying that ThT has multiple interaction modes during fibril formation.

Although a real-time NMR analysis of these interaction modes would certainly contribute to the elucidation of the detailed mechanism of amyloid formation, no in situ NMR study on amyloid formation has been reported so far. In particular, in the cases of natively folded amyloid-prone proteins such as polyubiquitin, β-lactoglobulin, and superoxide dismutase 1, additional mechanical forces and/or heat are required to initiate fibril formation [6,7,8,9]. However, no NMR method that can supply this kind of physical stimulation had been established. Recently, we established high-sensitivity Rheo-NMR spectroscopy [10], which is a novel NMR methodology to analyze protein samples under shear flow in situ. Since shear flow accelerates the fibril formation of amyloidogenic proteins [6,7], the Rheo-NMR methodology enables us to observe the fibril formation of amyloidogenic proteins in situ at the atomic level. Here, we applied Rheo-NMR to detect the interaction between ThT and amyloid fibrils in real time. We used a loop-truncated human superoxide dismutase 1 mutant (hSOD1^ΔIV,ΔVII,H46W^, hereafter: SOD1) as the amyloidogenic protein [11,12].

## 2. Results

### 2.1. Amyloid Fibril Formation of Superoxide Dismutase 1 (SOD1) in the Rheo-NMR Instrument

**Figure 1:**
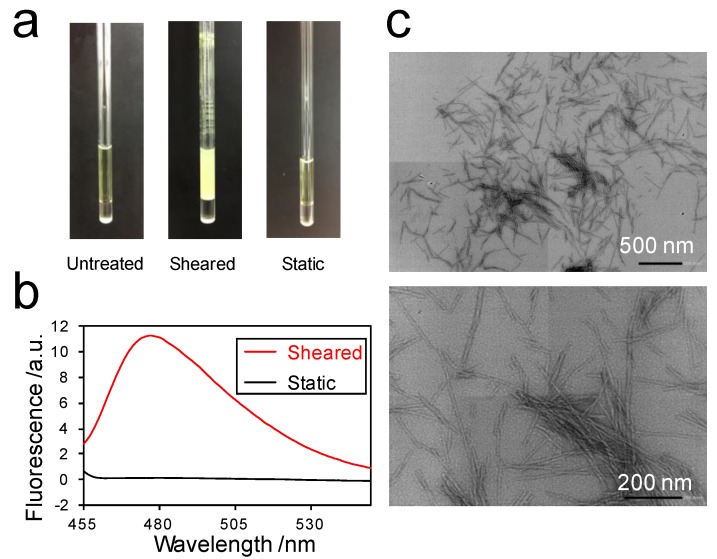
Superoxide dismutase 1 (SOD1) formed amyloid fibrils in the Rheo-NMR instrument: (**a**) Nuclear magnetic resonance (NMR) tubes containing the samples before (**left**) and after the Rheo-NMR measurements under sheared (**center**) and static conditions (**right**); (**b**) Thioflavin T (ThT) fluorescence spectra of sheared (red) and static (black) samples after the Rheo-NMR measurements for 48 h. a.u., arbitrary unit; (**c**) Transmission electron micrographs of SOD1 fibrils formed by shear flow. Scale bars, 500 nm (**upper** panel) and 200 nm (**lower** panel).

To investigate the interaction between ThT and the amyloid fibrils of SOD1, we first checked whether SOD1 forms amyloid fibrils in a solution containing ThT by using the Rheo-NMR instrument. Indeed, SOD1 formed aggregates by spinning the NMR tube at a frequency of 30 Hz for 48 h (Figure 1a, center). In stark contrast, we did not observe any aggregates under the static condition (Figure 1a, right), similar to an untreated solution (Figure 1a, left). In addition, the ThT fluorescence assay showed that the fluorescence intensity at 480 nm for the sheared sample was much higher than that of the static sample (Figure 1b). Transmission electron micrographs showed that the aggregates formed inside the Rheo-NMR instrument had typical amyloid fibril structures (Figure 1c). These results indicate that the application of shear flow resulted in the amyloid fibril formation of SOD1.

### 2.2. Decrease in Peak Volume of Soluble SOD1

**Figure 2:**
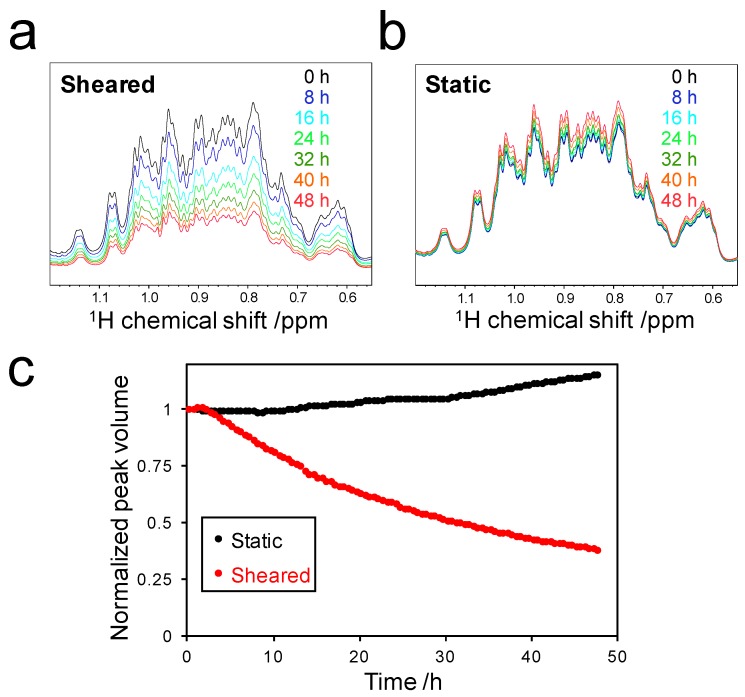
Decrease of soluble SOD1: Time-dependent spectral changes of methyl groups of soluble SOD1 under sheared (**a**) and static (**b**) conditions; (**c**) Real-time NMR profiles of the peak volume of the methyl groups under sheared (red) and static (black) conditions. The peak volume was calculated by the integration of the peaks in the ^1^H chemical shift range of 0.55 to 1.20 ppm and normalized by the initial peak volume.

To estimate amyloid formation during the Rheo-NMR measurements, we calculated the peak volume in the ^1^H chemical shift range of 0.55 to 1.20 ppm (Figure 2a,b). Almost all peaks in this region can be assigned to methyl groups of SOD1. Therefore, the total volume of these peaks reflects the concentration of soluble SOD1 species. The peak volume was found to be slightly increased due to the evaporation of water in the sample under the static condition (Figure 2c, black). On the other hand, the peak volume of SOD1 decreased under the sheared condition in a time-dependent manner (Figure 2c, red). This showed that monomeric SOD1 was converted into NMR-invisible high molecular weight species such as protofibrils and amyloid fibrils due to the application of shear flow. Interestingly, the peak volume decrease began approximately 2.5 h after the application of shear. This implied that the period from 0 to 2.5 h corresponds to the lag time of amyloid fibril formation [13]. After the lag time, the peak volume decreased exponentially and finally reached a value of approximately 38% of the initial peak volume. This indicated that the peak volume decrease of 62% was due to shear-induced oligomerization and/or amyloid fibril formation of SOD1. Considering water evaporation in the sample, the larger decrease in peak volume may be estimated, but it was difficult to quantify the actual values because water evaporation may depend on individual experimental conditions.

### 2.3. Detection of the Interaction between Thioflavin T (ThT) and SOD1 Fibrils

SOD1 formed fibrils during the Rheo-NMR measurements and the fluorescence of ThT increased due to the specific interaction with the SOD1 fibrils. To examine the chemical shift changes of ThT caused by its association with the fibrils, we monitored the chemical shift of ThT in real time during the fibril formation process. A previous study on the interaction between ThT and islet amyloid polypeptide (IAPP) fibrils [5] showed that the peak of the dimethylamino (DMA) group of ThT is most sensitive to the interaction with fibrils. Importantly, three different interaction modes are identified: weak interaction with fibrils, strong interaction with protofibrils, and strong interaction with fibrils. The DMA peak exhibits a small downfield shift when ThT weakly binds to fibrils. In the cases of strong interactions with protofibrils and fibrils, new DMA peaks at 3.38 and 3.65 ppm, respectively, can be detected. Based on these properties, we monitored the DMA signal during the fibril formation of SOD1, thereby obtaining real-time information on the interaction between ThT and the fibrils by Rheo-NMR measurements.

#### 2.3.1. Weak-Interaction-Induced Chemical Shift Changes of the Dimethylamino (DMA) Signal

**Figure 3:**
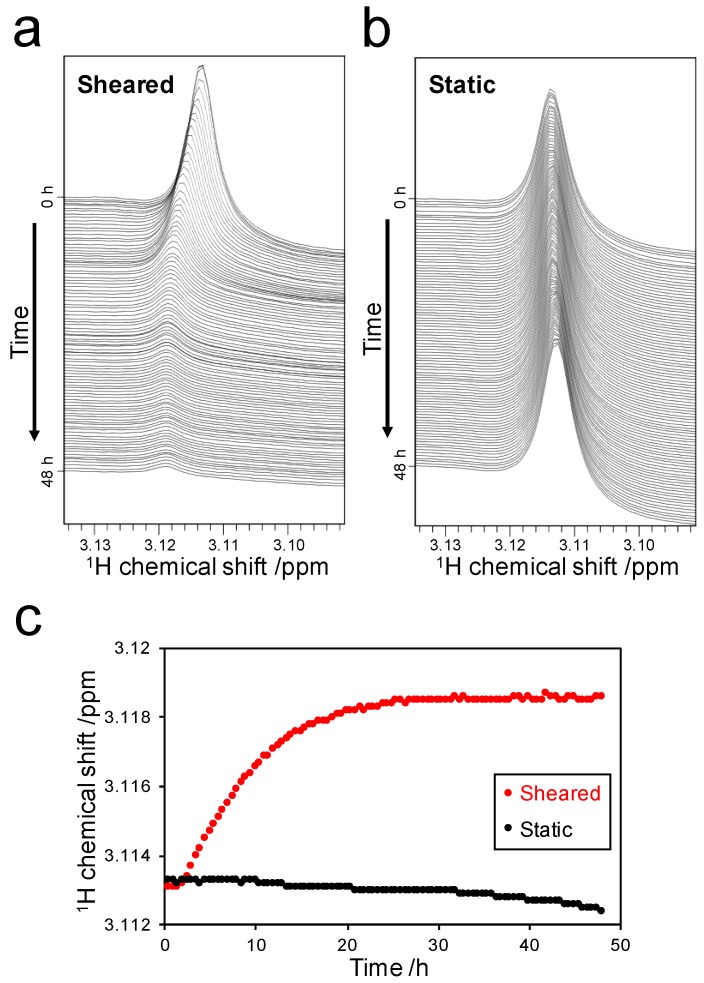
Detection of the weak interaction between ThT and the fibrils: Spectral changes in the range of 3.090 to 3.135 ppm under the sheared (**a**) and static (**b**) conditions. Spectra are displayed at intervals of 30 min; (**c**) Time-dependent chemical shift changes of the DMA peak under the sheared (red) and static (black) conditions.

The chemical shift of the DMA group was 3.113 ppm, which was not affected by the presence of monomeric SOD1 (Figure S1). Although the chemical shift did not change for 48 h under the static condition (Figure 3b), it was shifted under the sheared condition (Figure 3a). The chemical shift did not change in the first 2.5 h of the experiment, which was similar to the peak volume change during fibril formation (Figure 2c). However, the chemical shift exhibited marked changes in the period from 2.5 to 25 h. Finally, the chemical shift converged to a value of 3.119 ppm. This downfield shift of the DMA signal was similar to the previous report [5], indicating that a weak interaction of ThT with SOD1 fibrils caused the moderate downfield shift. In addition, line broadening of the DMA peak was observed during the process of fibril formation. This observation indicates that ThT bound to high molecular weight species, namely the amyloid fibrils.

#### 2.3.2. Detection of Strong Interactions between ThT and Fibrils

**Figure 4:**
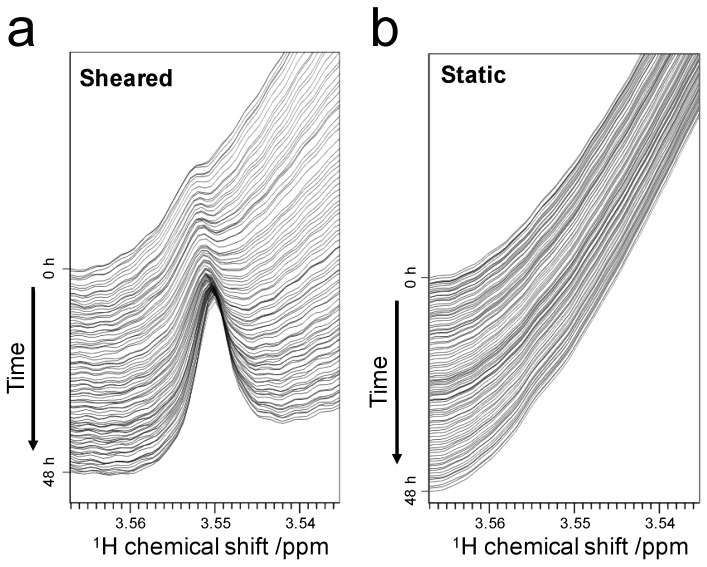
Appearance of a new peak in the process of fibril formation: Spectral changes during measurements under sheared (**a**) and static (**b**) conditions. Spectra are displayed at intervals of 30 min.

We detected that the weak interactions between ThT and SOD1 fibrils, observed on the DMA signal, was in fast exchange on the NMR timescale. In addition, a new peak derived from the DMA group was found under the sheared condition (Figure 4a). The chemical shift of the new peak was approximately 3.55 ppm, and its intensity increased in a fibril formation-dependent manner. The previous study showed that the DMA peak exhibited a pronounced downfield shift to 3.38 and 3.64 ppm in protofibril-rich and oligomer-rich IAPP samples, respectively [5]. The former shift to 3.38 ppm may arise from strong interaction with protofibrils, whereas the latter shift to 3.64 ppm might be caused when ThT is trapped in cavities on the fibril surface during the maturation of the fibrils [5]. By contrast, the chemical shift of the new peak observed in our experiment (3.55 ppm) did not correspond to the chemical shift of either case described above; therefore, it is difficult to assign the new peak to either protofibril- or oligomer-bound ThT DMA. These differences may result from the different measurement conditions, such as temperature and pH, and from the different types of amyloid proteins: SOD1 and IAPP. Interestingly, we observed that the intensity of the new peak increased over time (Figure 4a), indicating that the concentration of protofibrils or oligomers increased during fibril formation. In conclusion, we detected not only a weak interaction of ThT with mature amyloid fibrils, but also a strong interaction between ThT and premature amyloid species. We anticipate that these detailed observations will aid the understanding of the maturation process of amyloid fibrils.

## 3. Discussion

### 3.1. Stoichiometry of ThT-Fibril Binding

The peak corresponding to the DMA group exhibited chemical shift changes starting from 3.113 ppm, converging to a final value of 3.119 ppm (Figure 3c). This observation suggested that all ThT molecules bound to SOD1 fibrils. The chemical shift converged approximately 25 h after the initial application of shear (Figure 3c). At that time, the time-dependent peak volume changes of SOD1 showed that 44% of initial monomeric SOD1 contributed to fibril formation (Figure 2c). Although some of the ThT molecules contributed to the binding to protofibrils or oligomers, approximately 37.5 nmol of ThT bound to the fibrils, which consisted of 132 nmol of SOD1. This analysis suggests that one ThT molecule could bind to approximately 3.5 molecules of SOD1 in the fibril form.

### 3.2. Fluorescent-Dye-Based New Applications of Rheo-NMR

In this study, we used ThT as a fluorescent dye to detect fibril formation. We revealed the time evolution of the interaction between ThT and fibrils during amyloid formation. To date, other fluorescent dyes such as 1-anilinonaphthalene-8-sulfonic acid (ANS) [14,15], thiazin red [16], and Congo red [8,17] are widely used to analyze amyloid formation. In particular, ANS is a fluorescent probe that can detect prefibrillar species that are likely the most toxic species in amyloid pathology [14]. Therefore, the combined use of ANS and ThT—by monitoring both ANS and ThT signals by Rheo-NMR—will enable the detection of multiple fibrillation states in a quantitative manner in real time.

Importantly, our established methodology does not require any isotope labeling of protein samples. In most cases of protein NMR studies, the use of ^13^C- and/or ^15^N-isotope labeling is required to measure the NMR spectra of proteins. However, stable isotope compounds such as ^13^C-labeled glucose and ^15^N-labeled ammonium chloride are not inexpensive. Although deuterated buffer was used in this study to suppress the intense signals from buffer, phosphate buffer provides an inexpensive alternative. Phosphate groups do not have any detectable protons in NMR spectra, and therefore do not interfere with the NMR signals of ThT. As a result, ThT-based Rheo-NMR measurements using phosphate buffer would further reduce the experimental cost.

Furthermore, our Rheo-NMR methodology can be applied to high-molecular weight proteins. The high molecular weight of proteins causes severe line broadening and peak overlap, which makes it difficult to obtain structural information on the sample. In contrast, by using our Rheo-NMR methodology, kinetic information on the formation of amyloid species can be indirectly obtained from the signals of low molecular weight dyes. If the NMR signals of dyes overlap with protein signals, a T_2_ relaxation filter can be used to suppress the protein signals. This strategy enables the selective detection of dyes that have low molecular weight and a long T_2_ relaxation time. Moreover, our approach requires the acquisition of only one-dimensional NMR spectra, leading to short measurement times. This improves the time resolution of the fibril formation analysis, as compared with multi-dimensional NMR approaches. We anticipate that these methodological advantages will contribute to furthering the understanding of fibril formation of numerous amyloidogenic proteins.

## 4. Materials and Methods

### 4.1. Protein Expression and Purification

A pET3a plasmid encoding the H46W mutant of the loop-truncated human SOD1^ΔIV,ΔVII^ was transformed into Escherichia coli strain BL21(DE3). Cells were cultured in Luria-Bertani (LB) media and protein expression was induced by adding 0.5 mM isopropyl 1-thio-β-d-galactopyranoside (IPTG) to the media. Cells were disrupted by sonication and the supernatant was purified by ammonium sulfate precipitation and chromatography, as described previously [18]. The purity of the sample was checked by sodium dodecyl sulfate polyacrylamide gel electrophoresis (SDS-PAGE) (Figure S2).

### 4.2. Rheo-NMR Measurements

All Rheo-NMR measurements were performed at 298 K using a 600 MHz Bruker Avance spectrometer equipped with a 5-mm TXI triple resonance cryoprobe. Water-suppressed ^1^H NMR spectra were obtained by using the excitation sculpting pulse scheme [19]. The acquisition of one spectrum took 30 min. Continuous measurements were performed over a total time of 48 h. NMR samples were prepared at a concentration of 1.0 mM SOD1, 125 μM thioflavin T (Sigma-Aldrich, St. Louis, MO, USA), 100 μM sodium 2,2-dimethyl-2-silapentane-5-sulfonate (DSS, Tokyo Chemical Industry, Tokyo, Japan), 5% D_2_O, and 10 mM deuterated Bis-Tris HCl (Cambridge Isotope Laboratories, Cambridge, MA, USA) at pH 6.3. NMR measurements under the static condition were performed by not spinning the NMR tube. Rheo-NMR measurements under the sheared condition were performed by spinning the NMR tube at 30 Hz, corresponding to the shear rate of 434–811 s^−1^. The ^1^H chemical shift was calibrated using the methyl resonance of DSS. Data were processed and analyzed using TopSpin 3.5pl7 (Bruker BioSpin, Rheinstetten, Germany).

### 4.3. Thioflavin T Fluorescence Measurements

Thioflavin T fluorescence was measured on a FP-8300 instrument (JASCO, Tokyo, Japan). Thioflavin T emission was measured in the range of 455 to 550 nm by excitation at 440 nm. To prepare the samples for fluorescence measurements, the samples after Rheo-NMR measurements were diluted 100 times with 10 mM deuterated Bis-Tris HCl pH 6.3.

### 4.4. Transmission Electron Microscopy (TEM)

The suspension containing SOD1 fibrils after Rheo-NMR measurements was diluted 10 times for the preparation of the sample for TEM measurements. The sample was placed on a collodion-coated grid and stained with EM Stainer (Nisshin EM, Tokyo, Japan). Negatively stained TEM images were acquired on a JEM-1400 Plus instrument (JEOL, Tokyo, Japan).

## Supplementary Materials

Supplementary materials can be found at www.mdpi.com/1422-0067/18/11/2271/s1.

## Abbreviations

AD	Alzheimer’s disease
ALS	Amyotrophic lateral sclerosis
ANS	1-Anilinonaphthalene-8-sulfonic acid
DMA	Dimethylamino
DSS	Dimethyl-2-silapentane-5-sulfonate
IAPP	Islet amyloid polypeptide
IPTG	Isopropyl 1-thio-β-d-galactopyranoside
LB	Luria-Bertani
NMR	Nuclear magnetic resonance
PD	Parkinson’s disease
SDS-PAGE	Sodium dodecyl sulfate polyacrylamide gel electrophoresis
SOD1	Superoxide dismutase 1
TEM	Transmission electron microscopy
ThT	Thioflavin T

## References

[1] Ross C.A., Poirier M.A. (2004). Protein aggregation and neurodegenerative disease. Nat. Med..

[2] Conway K.A., Lee S.-J., Rochet J.-C., Ding T.T., Williamson R.E., Lansbury P.T. (2000). Acceleration of oligomerization, not fibrillization, is a shared property of both α-synuclein mutations linked to early-onset Parkinson’s disease: Implications for pathogenesis and therapy. Proc. Natl. Acad. Sci. USA.

[3] Cohen S.I., Linse S., Luheshi L.M., Hellstrand E., White D.A., Rajah L., Otzen D.E., Vendruscolo M., Dobson C.M., Knowles T.P. (2013). Proliferation of amyloid-β42 aggregates occurs through a secondary nucleation mechanism. Proc. Natl. Acad. Sci. USA.

[4] Naiki H., Higuchi K., Hosokawa M., Takeda T. (1989). Fluorometric determination of amyloid fibrils in vitro using the fluorescent dye, thioflavine T. Anal. Biochem..

[5] Robbins K.J., Liu G., Lin G., Lazo N.D. (2011). Detection of strongly bound thioflavin T species in amyloid fibrils by ligand-detected ^1^H NMR. J. Phys. Chem. Lett..

[6] Hill E.K., Krebs B., Goodall D.G., Howlett G.J., Dunstan D.E. (2006). Shear flow induces amyloid fibril formation. Biomacromolecules.

[7] Morimoto D., Walinda E., Fukada H., Sou Y.-S., Kageyama S., Hoshino M., Fujii T., Tsuchiya H., Saeki Y., Arita K. (2015). The unexpected role of polyubiquitin chains in the formation of fibrillar aggregates. Nat. Commun..

[8] Stathopulos P.B., Scholz G.A., Hwang Y.-M., Rumfeldt J.A.O., Lepock J.R., Meiering E.M. (2004). Sonication of proteins causes formation of aggregates that resemble amyloid. Protein Sci..

[9] Sasahara K., Yagi H., Naiki H., Goto Y. (2007). Heat-induced conversion of β2-microglobulin and hen egg-white lysozyme into amyloid fibrils. J. Mol. Biol..

[10] Morimoto D., Walinda E., Iwakawa N., Nishizawa M., Kawata Y., Yamamoto A., Shirakawa M., Scheler U., Sugase K. (2017). High-sensitivity Rheo-NMR spectroscopy for protein studies. Anal. Chem..

[11] Elam J.S., Taylor A.B., Strange R., Antonyuk S., Doucette P.A., Rodriguez J.A., Hasnain S.S., Hayward L.J., Valentine J.S., Yeates T.O. (2003). Amyloid-like filaments and water-filled nanotubes formed by SOD1 mutant proteins linked to familial ALS. Nat. Struct. Mol. Biol..

[12] Danielsson J., Kurnik M., Lang L., Oliveberg M. (2011). Cutting off functional loops from homodimeric enzyme superoxide dismutase 1 (SOD1) leaves monomeric β-barrels. J. Biol. Chem..

[13] Arosio P., Knowles T.P., Linse S. (2015). On the lag phase in amyloid fibril formation. Phys. Chem. Chem. Phys..

[14] Bolognesi B., Kumita J.R., Barros T.P., Esbjorner E.K., Luheshi L.M., Crowther D.C., Wilson M.R., Dobson C.M., Favrin G., Yerbury J.J. (2010). ANS binding reveals common features of cytotoxic amyloid species. ACS Chem. Biol..

[15] Baskakov I.V., Legname G., Baldwin M.A., Prusiner S.B., Cohen F.E. (2002). Pathway complexity of prion protein assembly into amyloid. J. Biol. Chem..

[16] Mena R., Edwards P., Pérez-Olvera O., Wischik C.M. (1995). Monitoring pathological assembly of tau and β-amyloid proteins in Alzheimer’s disease. Acta Neuropathol..

[17] McParland V.J., Kad N.M., Kalverda A.P., Brown A., Kirwin-Jones P., Hunter M.G., Sunde M., Radford S.E. (2000). Partially unfolded states of β2-microglobulin and amyloid formation in vitro. Biochemistry.

[18] Iwakawa N., Morimoto D., Walinda E., Sugase K., Shirakawa M. (2017). Backbone resonance assignments of monomeric SOD1 in dilute and crowded environments. Biomol. NMR Assign..

[19] Hwang T.-L., Shaka A.J. (1995). Water suppression that works. Excitation sculpting using arbitrary wave-forms and pulsed-field gradients. J. Magn. Reson. A.

