# Supplementary material for: Real-Time Observation of the Interaction between Thioflavin T and an Amyloid Protein by Using High-Sensitivity Rheo-NMR
Source: Int J Mol Sci. 2017 Oct 28;18(11):2271. doi: 10.3390/ijms18112271 (PMC5713241; doi:10.3390/ijms18112271)
Supplement: Supplementary file 1 [file ijms-18-02271-s001.pdf]

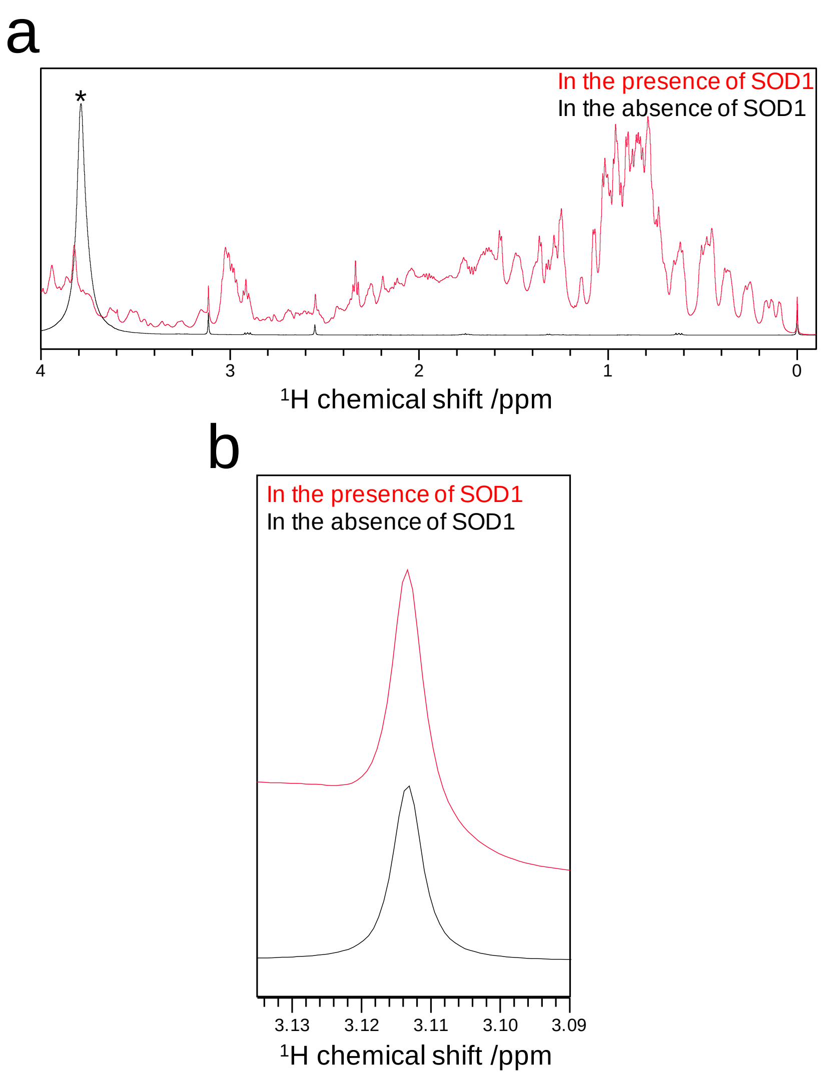

Figure S1. Comparison between the peaks of ThT in the absence and presence of monomeric SOD1: (**a**) ^{1}H NMR spectra of ThT in the range of -0.1 ppm to 4 ppm in the absence (black) and presence (red) of monomeric SOD1. The peak indicated by an asterisk in the spectrum is derived from Bis-Tris because the sample was dissolved in undeuterated Bis-Tris buffer for the NMR measurement of ThT without SOD1; (**b**) An enlarged view of the DMA peak in the absence (black) and presence (red) of monomeric SOD1.

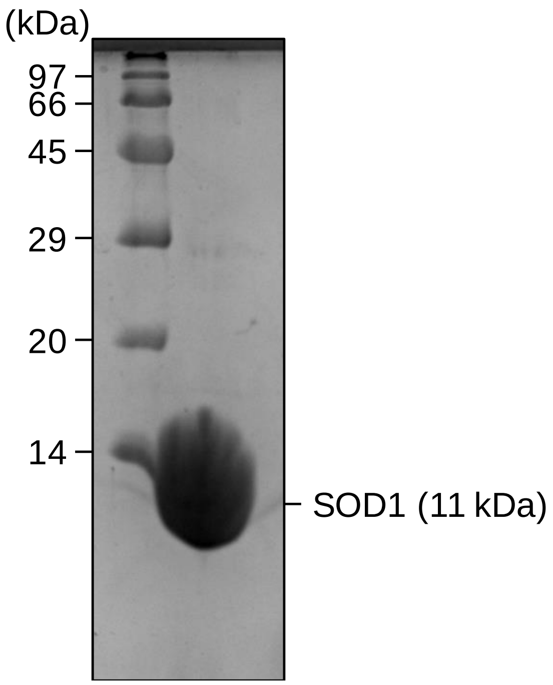

Figure S2. SDS-PAGE analysis of purified SOD1: Left lane, molecular weight marker; Right lane, SOD1 after purification by size-exclusion chromatography.
